# An integrative paradigm to impart quality to correlative science

**DOI:** 10.1186/1479-5876-8-26

**Published:** 2010-03-16

**Authors:** Michael Kalos

**Affiliations:** 1 Department of Pathology and Laboratory Medicine, University of Pennsylvania School of Medicine, Abramson Family Cancer Research Institute, University of Pennsylvania, 422 Curie Boulevard, BRBII/III, Philadelphia, PA 19104-4283, USA

## Abstract

Correlative studies are a primary mechanism through which insights can be obtained about the bioactivity and potential efficacy of candidate therapeutics evaluated in early-stage clinical trials. Accordingly, well designed and performed early-stage correlative studies have the potential to strongly influence further clinical development of candidate therapeutic agents, and correlative data obtained from early stage trials has the potential to provide important guidance on the design and ultimate successful evaluation of products in later stage trials, particularly in the context of emerging clinical trial paradigms such as adaptive trial design.

Historically the majority of early stage trials have not generated meaningful correlative data sets that could guide further clinical development of the products under evaluation. In this review article we will discuss some of the potential limitations with the historical approach to performing correlative studies that might explain at least in part the to-date overall failure of such studies to adequately support clinical trial development, and present emerging thought and approaches related to comprehensiveness and quality that hold the promise to support the development of correlative plans which will provide meaningful correlative data that can effectively guide and support the clinical development path for candidate therapeutic agents.

## Introduction

The primary objective of early stage clinical trials is to evaluate the safety of experimental therapeutic products. As a consequence, early stage trials have typically focused on the evaluation of novel experimental products on small cohorts of patients at late stages of disease, who have progressed through a series of prior treatments and are physiologically compromised in significant ways as a result of both disease status and prior treatment. Additionally, to minimize the potential for unanticipated toxicity issues, early stage trials typically evaluate novel therapeutic products at doses that are significantly lower than those predicted to have biological activity.

Correlative studies, which are common secondary objectives in clinical trials, can be described as covering two broad and related aspects of clinical trial research: the evaluation of markers associated with (i) positive clinical activity and (ii) product bioactivity and mechanism of action.

Since critical variables such as patient status, cohort size, and product dose are by intent sub-optimal, positive clinical activity is not commonly observed in early stage trials there is an inherent consequent inability to effectively identify and evaluate potential correlates of positive clinical activity. Nonetheless, the evaluation of correlates potentially associated with positive clinical activity is an important secondary objective of early stage trials, since any insights obtained through these analyses can help guide further clinical trial and correlative study development.

The evaluation of correlates for the biological activity and mechanism of action of the products is also potentially impacted by the safety-associated constraints of early clinical trials. The evaluation of correlates for product bioactivity is commonly accomplished through the evaluation of surrogate biological markers, functional or mechanistic, either directly associated with the product or that depend on the biological activity of the product. Any demonstration of product bioactivity during the early stage clinical trial process is an important indicator of successful delivery and bioactivity, and in the context of optimal biological dosing issues may help guide dosing schedules. This is particularly relevant for subsequent trial design, since the optimal biological dose (OBD) and dosing schedule of the product are likely to be distinct from the maximum tolerated dose (MTD). Early-stage insights into the biological effects of products are also important to appropriately and efficiently guide the further clinical development and validation as surrogate clinical biomarkers for product bioactivity and clinical efficacy. Finally, because at least a subset of candidate therapeutic products are likely to generate unanticipated biological effects, both positive and negative, it is also relevant to identify these effects in order to further characterize and address their impact on treatment outcome during later stage trials.

Robust and meaningful data about both product bioactivity and clinical activity are critical in the context of increasingly adopted adaptive trial design [1,2], which is based on the use of baeysian statistics to analyse data sets generated during the early stages of the clinical trial and in turn implement changes to fundamental clinical trial parameters such as primary endpoints, patient populations, cohort sizes and treatment arms, changes in statistical methodologies and changes in trial objectives [3,4].

Historically, the design of clinical correlative studies has been based on the scientific principles of hypothesis based experimentation which demands that research be based on specifically defined and testable hypotheses. The rationale and benefits of hypothesis-based research are clear: such research efforts are explicitly defined and focused, the ability to evaluate infrastructure and investigator capabilities is clear cut, and accountability for accomplishing specific goals can be objectively evaluated.

**Figure 1:**
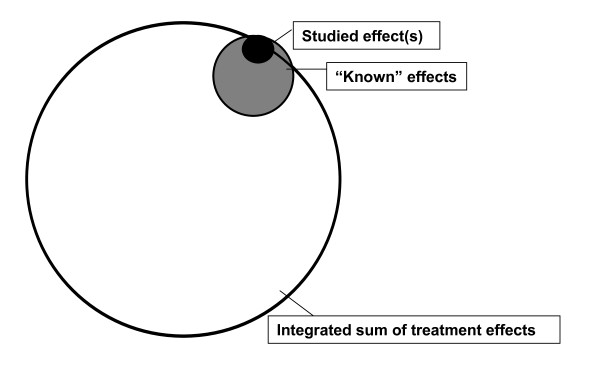
**The need for comprehensiveness in correlative studies**.

An unfortunate and unintended consequence of basing correlative studies primarily on principles of hypothesis based experimentation has been the establishment of a mind set that diminishes the value of hypothesis generating experimentation. Because it is impossible to have a comprehensive understanding of how candidate therapeutic agents impact patient biology from a whole systems perspective (Figure 1), our ability to define and implement the most appropriate correlative assays to evaluate candidate therapeutic agents is inevitably compromised, if driven only by hypotheses based on pre-existing biased views. Consequently, the concept of clinical correlative study design based solely or principally on hypothesis-based experimentation is fundamentally limiting, since it is destined to provide information on only a small subset of treatment associated events-those for which we have a-priori knowledge or insight. A complementary approach that ought to be considered in conjunction with hypothesis-based experimentation for clinical correlative studies involves the design and application of platforms and assays that are as broadly comprehensive as possible. Such an approach would allow for the identification and capture of a broad spectrum of data that have the potential to provide critical insight into the bioactivity and biological effects of the therapeutic moiety being studied, and also generate future testable hypotheses to be empirically evaluated in subsequent studies.

## Correlative studies-the past

Historically, five general principles have guided early-stage clinical correlative study design: (i) They have been dependent on the current state of knowledge about the agent studied and the target cell/tissue/organ (ii) They have been narrowly focused on parameters considered to be directly associated with clinical efficacy (iii) They have been based on the specific expertise and interest of the principal investigator (iv) They have been performed under general research laboratory standards in the laboratory of the clinical investigator directing the trial and (v) They have been budget constrained.

It is perhaps fair to state this approach for conducting correlative studies has failed, with precious few identifiable positive correlations established, even with low statistical significance, between disease impact and evaluated correlates, and an equal absence of systematic information about the bioactivity of evaluated products [5-9]. This is a remarkable, but nonetheless important statement, as it underlines the fact that our to-date approach for performing correlative studies suffers from significant limitations. The nature of these limitations and how we might move forward to overcome their impact on clinical trial analyses is the subject for the remainder of this review.

One obvious and significant reason for the to-date failure to identify meaningful correlates of treatment impact on disease and product bioactivity is related to the limitations discussed above (patient status, product dosing) imposed by the principal focus for early stage trials on safety. Beyond these important limitations, the general failure to identify biological correlates associated with positive outcomes in early-stage clinical trials can be attributed to two general possibilities: (i) the treatment has no potential to mediate positive clinical outcome and (ii) the treatment has the potential to mediate positive clinical outcomes, but those outcomes are a consequence of integration with secondary patient- and/or treatment-specific characteristics such as patient genetic background, genetic polymorphisms, and concomitant/prior treatments.

Since clinical trials are the end result of substantial research and development efforts that support the clinical evaluation of candidate products, it is reasonable to put forth the notion that in a reasonable number of cases there is an expectation for both product bioactivity and positive clinical activity. Beyond issues related to inadequate dosing, which can be evaluated through product bioactivity studies, this view would put forth the premise that the failure to identify meaningful biological correlates is a consequence of not looking for the correlations in an appropriate way. This can be interpreted as a failure to look with sufficient detail, in the appropriate tissue, at the appropriate time, and/or with the appropriate assay. One logical extension of this position is that for correlative studies to provide useful information it is critical that they be designed to be as comprehensive as possible. A necessary corollary position is to advocate for the more aggressive and committed funding of broadly focused and scientifically sound hypothesis generating studies to both complement existing- and initiate new-hypothesis testing studies.

With an understanding that future biological knowledge and insights will lead to currently unanticipated but potentially critical questions, an important corollary activity for each clinical study should be systematic and appropriate (i.e. high quality-based) banking of biological specimens (PBMC, marrow, tissue, tumor, lymph node, serum/plasma) for future evaluation. The importance of this endeavor cannot be overstated or substituted; simply put, in the absence of appropriate specimen banking, the potential to perform future correlative studies based on retrospectively identified and/or discovered relevant variables is irrevocably lost.

Practical limitations associated with the inability to sample most tissues at even a single time-point are a powerful impediment to being able to look for correlates in the appropriate way. To this end there is a pressing need to develop minimally invasive methodologies to procure microscopic samples from relevant tissue types as well as assays to evaluate these samples in a comprehensive manner. Some examples of novel assay platforms that offer the potential to evaluate very small samples in a more comprehensive manner are described in the following section of this review.

Finally, there has been an increasing appreciation for the need and benefits to conduct and evaluate early stage clinical studies in multi-institutional settings. Such efforts are accelerating the bench-to-bedside cycle of translational and clinical research by leveraging institutional-specific expertise and infrastructure within the consortia. A few examples of such multi-institutional consortia are government sponsored national and international clinical trial groups such as the Specialized Programs for Research Excellence (SPORE), the ISPY-2 adaptive clinical trial design effort in breast cancer, the Canadian Critical Care Trials Group, and the Ovarian Cancer Association Consortium (OCAC) [10-12].

## Comprehensiveness in correlative assays

One of the most exciting recent directions for correlative studies has been the development and implementation of strategies that address the need to evaluate samples in a more comprehensive manner. Broadly speaking, such methodologies are based on nucleic acid, flow cytometry, and biochemical platforms.

Nucleic acid array-based strategies have been applied in many cases to characterize the genotype [13,14] and molecular and proteomic expression phenotypes [13,15,16] of patient samples. A number of large multi-institutional consortia-based efforts supported through programs such as the SPORE are underway to support large scale clinical molecular profiling efforts and such efforts are beginning to provide valuable insights with regard to correlates of efficacy in various clinical settings [10].

Flow cytometry-based strategies have played a prominent role in clinical correlative studies for a number of years. The advent of multi-laser flow cytometers capable of "routinely" detecting upwards of 12 distinct fluorochromes has revolutionized the ability to apply flow cytometry to clinical correlative studies. Cell subsets can now be identified on the basis of surface markers, characterized in terms of their activation and/or differentiation status, and studied in terms of their effector functions by measuring intracellular cytokines, detecting protein phosphorylation status of signal transduction mediators or using functional assays [17-20]. The Roederer group initially and others subsequently have described the concept of polyfunctional T cells and protective immunity has been shown to be associated with T cells that integrate multiple effector functions[21,22]. To accommodate the need to evaluate in a relational manner the large data sets derived from these experiments specialized programs and algorithms have been generated to allow for analysis of data [23].

A number of platforms have been recently established that allow for the simultaneous evaluation of multiple analytes (multiplex analyses) in samples. Such platforms include the Luminex bead array [24], the cytokine bead array [25], and Meso-scale discovery sign arrays[26], and based on these platforms commercially available panels are now available to quantify cytokines/chemokines/growth factors potentially associated with numerous disease conditions and indications. Multiplex assays have been developed to allow for quantification of protein and phosphoprotein species in biological fluids such as serum, plasma, follicular fluid, and CSF, as well as tissue culture medium [24,27-29], as well as nucleic acids isolated directly from tissue samples[30,31].

Novel platforms based on newly developed technologies are at the cusp of revolutionizing our ability to be comprehensive in correlative study design. Some examples of these exciting advances include the development of methodologies to couple antibodies to elemental isotopes combined with the use of inductively coupled plasma mass spectrometry (ICP-MS) to detect and quantify the antibodies in atomized and ionized samples [32], the conjugation of antibodies to single strand DNA oligomers (DEAL-DNA Encoded Antibody Libraries) that can bind to nucleic acids or proteins in biological samples and the use of microfluidics-based instrumentation to interrogate individual cell samples in a multiplex manner [33], and the development of emulsion PCR coupled with microfluidics to simultaneously perform and collect data on thousands of PCR reactions in parallel [34].

As correlative platforms which generate more comprehensive data sets are implemented, it will be critical to take into account the strong possibility that identification of relevant correlates will need to rely on systems biology-based analyses to reveal multi-factorial signatures that correlate with treatment outcome and bioactivity. Such systems biology-based approaches will require integration of data generated from multiple and distinct correlative assay platforms, with data collected in both research and clinical laboratories. With this in mind, one important issue that needs to be adequately addressed is the need for appropriate infrastructure to catalogue and analyze the data. Specific strategies for data collection, annotation, storage, statistical analysis, and interpretation should be established up front to guide such studies. In this regard, establishment of common or relateable annotation schemes for data files will be essential to allow for implementation of the complex algorithms necessary to identify the biological signatures which correlate with disease impact. As discussed in more detail below, efforts such as the MIBBI project are underway to systematize data collection, annotation, storage, and analysis.

It is essential to keep in mind the high probability for a low clinical response rate in early stage trials. As discussed above, it is imperative to integrate in the correlative design process studies to evaluate product bioactivity, ideally by measuring direct impact on the molecular target of the treatment, so that correlates of disease impact can be retrospectively evaluated in the patient cohorts where the treatment has impacted the defined target.

A challenge for the correlative community is the inherent complication of utilizing new and non-validated platforms and assays to generate data sets which reveal novel multi-factorial signatures that correlate with treatment outcome or product bioactivity. It is important to ensure that such assays are performed with stringent performance controls for both the instruments and the assay to assure reproducibility of the data. The implementation of quality at this level will enable the optimal integration and interpretation of these data sets, and will also establish the foundations for qualification and validation of both the assays and the multi-factorial signatures prior to use in correlative analyses for subsequent trials.

## Principles of quality in correlative studies

In the context of this discussion, we will define quality as the implementation of laboratory procedures, infrastructure, and an organizational mindset that enable the generation of scientifically data that are objectively rigorous and sound.

Since objective standards do not exist for defining quality in basic research laboratory operations, the implementation of principles of quality for correlative studies performed in these laboratories has been dependent on an ad-hoc understanding by individual laboratories of what quality means and how it can be achieved. A consequence of this fact has been a disparity in the application of principles of quality across laboratories, and an implementation of rigorous standards of laboratory operation for instrument use, assay performance and analysis in only a subset of laboratories. Perhaps predictably, this has resulted in a disparity in data quality across laboratories, and an inability of the larger scientific community to readily interpret correlative data and move the field forward in the most productive fashion. Recently published results from early stage proficiency panels sponsored by the CVC/CRI discussed later in this document provide a clear example for both the disparity in quality of data across basic research laboratories and also clearly demonstrate the existence of research level correlative laboratories that generate reproducible and high quality data sets.

The engagement and continued participation of professional statistical support is an essential component of the quality process in correlative studies, and the input of biostatisticians is critical at all stages of the assay process, beginning with assay development all the way through the assay qualification/validation process and subsequent performance. To this end, specific effort should be put forth to educate both biostatisticians to ensure that they have a concrete understanding of the scientific, biological, and clinical questions being studied, and researchers to ensure that they have a concrete understanding of the potential constraints and limitations imposed on the assays and the clinical study by the requirements needed to generate data sets that are statistically meaningful. Furthermore, the active and continued participation of biostatistical support in the clinical trial design is critical to allow for appropriate patient cohort sizes to evaluate proposed hypotheses.

For correlative studies to provide meaningful and readily interpretable information it is critical that they be conducted in a manner that is as scientifically sound as possible. In particular, correlative assays should (i) measure what they claim to measure, (ii) be quantitative and reproducible and (iii) produce results that are statistically meaningful. In other words, correlative studies need to be performed using assays that are at a minimum qualified, and more appropriately validated for their performance characteristics.

The principles for assay qualification and validation have been developed in the context of chemical and microbiological/ligand based assays, in relatively well defined in-vitro systems under conditions where experimental parameters and assay variables can be defined relatively rigorously. In the context of biological systems, the concept of assay qualification and/or validation is complicated by the inherent undefined complexity and variability of sample source and composition. This complexity and variability has been used to support the position that assay qualification and validation are not tenable objectives for most biological assays. An opposing view advocated here is that it is precisely because biological assays are complex and variable that all reasonable efforts must be made to conform as much as possible to principles of quality. This position has merit even in the context of trials where candidate products do not demonstrate efficacy, since information generated from comprehensive and quality correlative studies has the potential to reveal mechanistic reasons for the lack of efficacy that can in principle be addressed with additional product development efforts and subsequent trials.

## Qualified and Validated Assays

A Qualified Assay is one for which the conditions have been established under which, provided it is performed under the same conditions each time, the assay will provide meaningful (i.e. accurate, reproducible, statistically supported) data. Since the term "meaningful data" in itself is subjective and there are no set guidelines for qualifying assays, assay qualification is a subjective and therefore from a quality perspective difficult process. Qualified assays have no predetermined performance specifications (i.e. no pass/fail parameters) and are often used to determine the performance specifications critical to establishing validated assays.

Straight forward examples of applying the assay qualification process to biological assays can be derived from experiments designed to define the optimal parameters for assay performance. For example, in the case of proliferation assays, experiments to determine the optimal ratio and range of antigen presenting:effector cells, culture medium, and time of culture, and in the case of Q-PCR assays, experiments to determine the optimal amplification conditions (primer concentration, input nucleic acid, annealing and extension times and temperatures) are all experiments that identify assay conditions which allow for the ability to obtain reproducible and meaningful data.

Although there is no requirement to utilize established Standard operating Protocols (SOP) when performing qualified assays, it is an excellent idea to do so. Finally, because the acceptance of data from a qualified assay depends on operator judgment, qualified assays should only be run by highly experienced laboratory staff.

Validated assays are assays for which the conditions (specifications) have been established to assure that the assay is working appropriately every time it is run. Standard Operating Protocols (SOP) are absolutely required for validated assays and the specifications (also known as assay pass/fail parameters), are pre-established as part of the validation process and must be met at every run. Validated assays almost always require the development of reference samples (positive and negative), as well as the establishment of standard curves that are used to derive numerical data for test samples.

A guidance document for the validation of bioanalytical assays is available through the FDA website http://www.fda.gov/cder/guidance/. Although this document has been prepared to support validation of chemical and microbiological/ligand based assays, it provides an excellent foundation to support the development of validation plans for biological assays.

As detailed in the guidance document, a validation plan needs to address and if feasible evaluate the following parameters with statistical significance:

1. Specificity/selectivity: The ability to differentiate and quantify the test article in the context of the bioassay components.

2. Accuracy: The closeness of the test results to the true value. Often this is very difficult to ascertain for biological assays as it requires an independent true measure of this variable.

3. Precision (intra- and inter-assay). How close values are upon replicate measurement, performed either within the same assay or in independent assays.

4. Calibration/standard curve (upper and lower limits of quantification). The range of the standard curve that can be used to quantify test values. This range can be (and often is) different from the limit of detection (see below).

5. Detection limit. The lowest value that can be detected above the established negative or background value.

6. Robustness. How well the assay transfers to another laboratory and/or another instrument within the same laboratory.

### The assay validation process

The assay validation process involves a series of discrete and formal steps that are initiated and completed with the generation of formal documents:

(i) The initial process is to define the assay (what will it measure, how it will be measured), and how each of the validation parameters will be addressed and evaluated. It is possible that for a particular assay, one or more of the validation parameters will not be relevant or addressable; this is acceptable but the reasons for this must be formally described. This process initiates with the creation of an initial assay validation master plan document within which are described the purpose and design of the validation studies and how each of the parameters will be addressed, and is completed with the creation of a pre-validation proposal document used in following.

(ii) The pre-validation stage establishes the parameters for qualifying the assay by performing a series of exploratory and optimization experiments that address each of the validation parameters. The end result of the pre-validation stage is a formal report which describes and summarizes the results of the studies, and establishes specification and acceptance criteria as well as a validation plan for specific experiments to be performed to validate the established criteria. For data sets that conform to Gaussian distributions, determination of the 95% prediction interval values can be a reasonable mechanism to establish assay specifications and acceptance criteria.

(iii) The validation stage involves conducting a series of experiments, designed with statistical input, to evaluate whether the specification values established during the pre-validation stage can be met. The validation stage is preceded by the creation of a document that describes a formal validation plan where validation experiments, specification values tested, and statistical analyses are defined a-priori. A method can fail all or part of the validation process; that is to say validation studies may reveal that the pre-established acceptance criteria cannot be met. If this occurs, the failure needs to be investigated and cause assigned. If failure is determined to reflect a deficiency in the protocol employed, the protocol may be revised but the entire validation process should be repeated. If failure is attributed to improper assessment of acceptance criteria the criteria can be reassigned and those specific validation studies need be repeated.

(iv) Once the validation studies are completed, a formal validation report is compiled, and assay SOP and worksheets are completed and released for use.

A summary Table that describes and compares assay qualification and assay validation can be found in Appendix 1, while a summary Table that describes an overview of the assay validation process can be found in Appendix 2.

### Imparting quality to biological assays

As discussed above, assay validation has been most often implemented in the context of bioanalytical assays with well defined analytes and sample matrixes. On the other hand, biological assays commonly involve evaluation of materials obtained from patients and are complicated by the absence of detailed and specific information for both the analyte as well as the biological matrix. Some understanding for the difficulties associated with imparting quality to biological assays might be understood from the following examples: Assessment of accuracy requires knowledge about the "true value" for what is being measured which is often not available for the analyte under evaluation. Patient whole blood samples obtained through a time course can be remarkably different in cellular, cytokine and hormonal composition with a consequent variability dramatically affecting the nature of the matrix for the analyte under evaluation. Changes in T cell avidity due to changes in activation status may have profound and entirely unanticipated consequences on the specificity, accuracy, sensitivity, or robustness of a biological assay. Thus, depending on the biological assay, it may not be possible to validate one or more of the above described validation parameters and establish a fully validated assay. Nonetheless, and perhaps because of this complexity, it is imperative that biological assays be established and performed with a vigilance for imparting rigorous quality support.

The statistical underpinnings for validated assays need to be established on an assay-specific basis and with formal statistical input from a bona-fide statistician, both for design of the validation plan and also to provide appropriate guidance for defining acceptable variability for the assays.

Some general guidelines to help impart quality on biological assays include:

(i) Establish SOP for the assays and instrumentation and limit assays to trained users and operators. (ii) Evaluate parameters using multiple sources of biological material, ideally obtained under conditions similar to the experimental. (iii) Develop reference cell lines (positive and negative), and establish dedicated master cell line stocks for all reference cells. (iv) Establish statistically supported quality parameters for the reference cell lines; these parameters can be use as pass/fail criteria for the assay performance.

## Establishing quality in correlative laboratories

Presently there is no formal requirement (for example GMP/GLP/cGLP/CLIA/CAP/etc.) for quality certification of laboratories that perform correlative assays. With this in mind, and with an appreciation for the fact that formal validation is often not feasible for biological assays, it is worthwhile to discuss a practical approach for how to establish quality in correlative labs, particularly in an era of dwindling funding for available research.

Perhaps the most important component to establish quality in correlative laboratories is to explicitly support a laboratory environment that supports quality. To that end, specific guidelines might include: (a) Develop SOP for all laboratory procedures and processes, including not only assay methodologies but also, sample receipt, processing, and storage, personnel training, equipment maintenance/calibration, data management, and repository activities. (b) Invest the time and funds to develop qualified/validated assays (c) Establish reference standards whenever possible and creating master lots and/or cell banks for all standards.

The appreciation for the need to impart more objective quality standards to correlative studies has been gaining momentum in the broader correlative research community, and a number of organizations have sponsored and/or supported consortia to establish and support quality in correlative studies. In some cases, exemplified by the efforts of the HIV clinical Trial network (HVTN), the primary purpose of the consortium efforts were to enable multi-national clinical correlative studies to be performed in a standardized manner and with quality infrastructure. For other consortia, such as the Cancer Vaccine Consortium/Cancer Research Institute (CVC/CRI) and the Association for the Immunotherapy of Cancer (C-IMT) which have each sponsored proficiency and harmonization panels, the primary purpose is to identify the assay variables that are associated with assay performance variability and provide guidelines for improving the quality of immune correlative assays. The initial results from some of these harmonization efforts have recently been published [35,36]; importantly these reports empirically demonstrate the need to establish quality infrastructure in correlative labs since most parameters identified to impact assay performance are specifically related to the establishment and implementation of quality-enabling infrastructure. An additional message that these initial proficiency panels reinforce is that objective quality is not to be assumed, and that it is critical to objectively evaluate, establish, and maintain quality infrastructure in correlative laboratories.

The concept of assay harmonization across laboratories that perform the same general correlative assay is one that merits consideration particularly for early-stage clinical trials, since the end product of the harmonization process is the establishment of laboratory equipment- and infrastructure-specific assay protocols which allow for the generation of data sets that are directly comparable across laboratories.

The MIBBI (Minimum Information about Biological and Biomedical Investigations) project [37] represents another effort to impart quality in biological assays. MIBBI associated efforts involve the establishment, through transparent and open community participation, of minimum assay-related information checklists and web-based databases for entry and access to the information. MIBBI reporting guidelines address two related and important issues for correlative science: i. the need to be able to critically assess the quality infrastructure associated with published data sets and ii. The need to establish common or relatable terminology for reporting and annotating the data. MIBBI guidelines have now been published for a number of fields including microarray and gene expression, proteomics, genotyping, flow cytometry, cellular assays [37] as well as T cell and other immune assays[38].

Another example of efforts to bring quality into immune correlative studies is the establishment of nationally sponsored immune monitoring program to support harmonized and quality immune monitoring program for clinical trials, as exemplified by the Canadian government-sponsored immune monitoring program http://www.niml.org. Such paradigm-shifting efforts facilitate the harmonized and/or standardized application of correlative assays across multiple clinical centers, and also set the stage for the effective sharing of resources such as reagents, assay protocols/SOP and clinical samples to allow for a more harmonized and systematic analysis of clinical samples.

## Conclusions

Since correlative studies are the primary mechanism through which insights can be obtained about the efficacy and biological effects of novel therapeutics, how we perform correlative studies is critical for the effective evaluation and development of clinical trials, to justify the years of preclinical and clinical efforts and costs, as well as patient time and commitment to the clinical trial process.

It has become apparent that correlative studies which are performed on the basis of narrowly defined parameters and without the support of quality laboratory infrastructure are extremely unlikely to yield meaningful information about the efficacy of novel therapeutic products. With that in mind there is considerable scientific and practical rationale to design correlative studies that are as comprehensive as possible, and performed to the highest possible scientific standard. While well performed correlative studies are critical in early stage trials that show evidence of efficacy and product bioactivity so that efficacy and product biomarkers can be identified and further developed in later stage trials, and are also important in early stage trials that do not show evidence of efficacy since the correlative studies can potentially reveal reasons for the failure of the product that can be addressed in further product development and if appropriate.

From both a scientific and financial perspective there is significant rationale and justification for the support of dedicated facilities with quality systems in place to perform comprehensive correlative studies. The implementation of quality- and comprehensive study-enabling infrastructure in dedicated laboratories that perform correlative studies provides for a rational expectation to be able to generate more relevant and informative data sets to interpret and guide product development through the clinical trial process.

## Competing interests

The authors declare that they have no competing interests.

## Appendix 1: Assay Qualification vs. Assay Validation

### Assay Qualification process

Establishes that an assay will provide meaningful data under the specific conditions used

• No predetermined performance specifications

• No set guidelines for qualifying assay

• Used to determine method performance capabilities, including validation parameters

### Qualified assay

• Approved Standard Operating Procedure is desirable, but not required; however, procedures must be documented adequately

• Assay should be run by highly qualified and experienced staff

• Assay validity is based on operator judgment

### Assay Validation Process

Establishes conditions and specifications to assure that the assay is working appropriately every time it is run

• Specifications established prior to validation

• Specifications must be met at every run

• Method can fail validation. If it does fail, an investigation must be conducted and cause assigned

### Validated assay

• Has established conditions (specifications) to assure that the assay is working appropriately every time it is run

• Standard Operating Procedure absolutely required

• Specifications must be met in every run

• Assay validity determined by pre-established assay criteria

## Appendix 2: Assay Validation

### Assay Validation Overview

Define assay: Define what will assay measure and how will it be measured

Define how each of the validation parameters will be evaluated with statistical significance

• Specificity

• Accuracy

• Precision (inter- and intra-assay)

• Calibration/standard curve (upper and lower limits of quantification)

• Detection limit

• Robustness

### Validation process

1. Pre-validation stage

- Perform exploratory and optimization procedures

2. Establish and define assay specifications

- Compile pre-validation report

- Compose validation plan that includes specification and acceptance criteria

3. Perform validation studies. These studies need to meet specification values

4. Compile validation report

5. Complete Standard Operating Procedure and worksheets

## References

[B1] Hoos A Parmiani G Hege K Sznol M Loibner H Eggermont A Urba W Blumenstein B Sacks N Keilholz U A clinical development paradigm for cancer vaccines and related biologics J Immunother 2007 30 1 15 10.1097/01.cji.0000211341.88835.ae 17198079

[B2] Chow SC Chang M Adaptive design methods in clinical trials - a review Orphanet J Rare Dis 2008 3 11 10.1186/1750-1172-3-11 18454853 PMC2422839

[B3] Berry DA Adaptive trial design Clin Adv Hematol Oncol 2007 5 522 524 17679925

[B4] Biswas S Liu DD Lee JJ Berry DA Bayesian clinical trials at the University of Texas M. D. Anderson Cancer Center Clin Trials 2009 6 205 216 10.1177/1740774509104992 19528130 PMC2913209

[B5] Jain RK Duda DG Willett CG Sahani DV Zhu AX Loeffler JS Batchelor TT Sorensen AG Biomarkers of response and resistance to antiangiogenic therapy Nat Rev Clin Oncol 2009 6 327 338 10.1038/nrclinonc.2009.63 19483739 PMC3057433

[B6] Le TC Vidal L Siu LL Progress and challenges in the identification of biomarkers for EGFR and VEGFR targeting anticancer agents Drug Resist Updat 2008 11 99 109 10.1016/j.drup.2008.04.001 18515176

[B7] Lee JW Figeys D Vasilescu J Biomarker assay translation from discovery to clinical studies in cancer drug development: quantification of emerging protein biomarkers Adv Cancer Res 2007 96 269 298 10.1016/S0065-230X(06)96010-2 17161683

[B8] Sarker D Workman P Pharmacodynamic biomarkers for molecular cancer therapeutics Adv Cancer Res 2007 96 213 268 10.1016/S0065-230X(06)96008-4 17161682

[B9] Sathornsumetee S Rich JN Antiangiogenic therapy in malignant glioma: promise and challenge Curr Pharm Des 2007 13 3545 3558 10.2174/138161207782794130 18220791

[B10] Barker AD Sigman CC Kelloff GJ Hylton NM Berry DA Esserman LJ I-SPY 2: an adaptive breast cancer trial design in the setting of neoadjuvant chemotherapy Clin Pharmacol Ther 2009 86 97 100 10.1038/clpt.2009.68 19440188

[B11] Fasching PA Gayther S Pearce L Schildkraut JM Goode E Thiel F Chenevix-Trench G Chang-Claude J Wang-Gohrke S Ramus S Role of genetic polymorphisms and ovarian cancer susceptibility Mol Oncol 2009 3 171 181 10.1016/j.molonc.2009.01.008 19383379 PMC5527888

[B12] Marshall JC Cook DJ Investigator-led clinical research consortia: the Canadian Critical Care Trials Group Crit Care Med 2009 37 S165 S172 10.1097/CCM.0b013e3181921079 19104219

[B13] Coco S Tonini GP Stigliani S Scaruffi P Genome and transcriptome analysis of neuroblastoma advanced diagnosis from innovative therapies Curr Pharm Des 2009 15 448 455 10.2174/138161209787315792 19199972

[B14] Shen Y Wu BL Microarray-based genomic DNA profiling technologies in clinical molecular diagnostics Clin Chem 2009 55 659 669 10.1373/clinchem.2008.112821 19233918

[B15] Lionetti M Agnelli L Mosca L Fabris S Andronache A Todoerti K Ronchetti D Deliliers GL Neri A Integrative high-resolution microarray analysis of human myeloma cell lines reveals deregulated miRNA expression associated with allelic imbalances and gene expression profiles Genes Chromosomes Cancer 2009 48 521 531 10.1002/gcc.20660 19306352

[B16] Raj A van OA Single-molecule approaches to stochastic gene expression Annu Rev Biophys 2009 38 255 270 10.1146/annurev.biophys.37.032807.125928 19416069 PMC3126657

[B17] Nomura L Maino VC Maecker HT Standardization and optimization of multiparameter intracellular cytokine staining Cytometry A 2008 73 984 991 18612990 10.1002/cyto.a.20602

[B18] Nolan JP Yang L The flow of cytometry into systems biology Brief Funct Genomic Proteomic 2007 6 81 90 10.1093/bfgp/elm011 17611236

[B19] Hale MB Nolan GP Phospho-specific flow cytometry: intersection of immunology and biochemistry at the single-cell level Curr Opin Mol Ther 2006 8 215 224 16774041

[B20] Seder RA Darrah PA Roederer M T-cell quality in memory and protection: implications for vaccine design Nat Rev Immunol 2008 8 247 258 10.1038/nri2274 18323851

[B21] Makedonas G Betts MR Polyfunctional analysis of human t cell responses: importance in vaccine immunogenicity and natural infection Springer Semin Immunopathol 2006 28 209 219 10.1007/s00281-006-0025-4 16932955

[B22] Precopio ML Betts MR Parrino J Price DA Gostick E Ambrozak DR Asher TE Douek DC Harari A Pantaleo G Immunization with vaccinia virus induces polyfunctional and phenotypically distinctive CD8(+) T cell responses J Exp Med 2007 204 1405 1416 10.1084/jem.20062363 17535971 PMC2118607

[B23] Petrausch U Haley D Miller W Floyd K Urba WJ Walker E Polychromatic flow cytometry: a rapid method for the reduction and analysis of complex multiparameter data Cytometry A 2006 69 1162 1173 17089357 10.1002/cyto.a.20342

[B24] Nolen BM Marks JR Ta'san S Rand A Luong TM Wang Y Blackwell K Lokshin AE Serum biomarker profiles and response to neoadjuvant chemotherapy for locally advanced breast cancer Breast Cancer Res 2008 10 R45 10.1186/bcr2096 18474099 PMC2481492

[B25] Morgan E Varro R Sepulveda H Ember JA Apgar J Wilson J Lowe L Chen R Shivraj L Agadir A Cytometric bead array: a multiplexed assay platform with applications in various areas of biology Clin Immunol 2004 110 252 266 10.1016/j.clim.2003.11.017 15047203

[B26] Marchese RD Puchalski D Miller P Antonello J Hammond O Green T Rubinstein LJ Caulfield MJ Sikkema D Optimization and validation of a multiplex, electrochemiluminescence-based detection assay for the quantitation of immunoglobulin G serotype-specific antipneumococcal antibodies in human serum Clin Vaccine Immunol 2009 16 387 396 10.1128/CVI.00415-08 19158284 PMC2650878

[B27] Ledee N Lombroso R Lombardelli L Selva J Dubanchet S Chaouat G Frankenne F Foidart JM Maggi E Romagnani S Cytokines and chemokines in follicular fluids and potential of the corresponding embryo: the role of granulocyte colony-stimulating factor Hum Reprod 2008 23 2001 2009 10.1093/humrep/den192 18503053

[B28] Choi C Jeong JH Jang JS Choi K Lee J Kwon J Choi KG Lee JS Kang SW Multiplex analysis of cytokines in the serum and cerebrospinal fluid of patients with Alzheimer's disease by color-coded bead technology J Clin Neurol 2008 4 84 88 10.3988/jcn.2008.4.2.84 19513308 PMC2686871

[B29] Pelech S Tracking cell signaling protein expression and phosphorylation by innovative proteomic solutions Curr Pharm Biotechnol 2004 5 69 77 10.2174/1389201043489666 14965210

[B30] Bortolin S Multiplex genotyping for thrombophilia-associated SNPs by universal bead arrays Methods Mol Biol 2009 496 59 72 full_text 18839105 10.1007/978-1-59745-553-4_6

[B31] Dunbar SA Applications of Luminex xMAP technology for rapid, high-throughput multiplexed nucleic acid detection Clin Chim Acta 2006 363 71 82 10.1016/j.cccn.2005.06.023 16102740 PMC7124242

[B32] Ornatsky OI Kinach R Bandura DR Lou X Tanner SD Baranov VI Nitz M Winnik MA Development of analytical methods for multiplex bio-assay with inductively coupled plasma mass spectrometry J Anal At Spectrom 2008 23 463 469 10.1039/b710510j 19122859 PMC2600572

[B33] Bailey RC Kwong GA Radu CG Witte ON Heath JR DNA-encoded antibody libraries: a unified platform for multiplexed cell sorting and detection of genes and proteins J Am Chem Soc 2007 129 1959 1967 10.1021/ja065930i 17260987 PMC3677962

[B34] Zimmermann BG Grill S Holzgreve W Zhong XY Jackson LG Hahn S Digital PCR: a powerful new tool for noninvasive prenatal diagnosis? Prenat Diagn 2008 28 1087 1093 10.1002/pd.2150 19003785

[B35] Britten CM Janetzki S Ben-Porat L Clay TM Kalos M Maecker H Odunsi K Pride M Old L Hoos A Harmonization guidelines for HLA-peptide multimer assays derived from results of a large scale international proficiency panel of the Cancer Vaccine Consortium Cancer Immunol Immunother 2009 58 1701 13 10.1007/s00262-009-0681-z 19259668 PMC2714899

[B36] Janetzki S Panageas KS Ben-Porat L Boyer J Britten CM Clay TM Kalos M Maecker HT Romero P Yuan J Results and harmonization guidelines from two large-scale international Elispot proficiency panels conducted by the Cancer Vaccine Consortium (CVC/SVI) Cancer Immunol Immunother 2008 57 303 315 10.1007/s00262-007-0380-6 17721781 PMC2150634

[B37] Taylor CF Field D Sansone SA Aerts J Apweiler R Ashburner M Ball CA Binz PA Bogue M Booth T Promoting coherent minimum reporting guidelines for biological and biomedical investigations: the MIBBI project Nat Biotechnol 2008 26 889 896 10.1038/nbt.1411 18688244 PMC2771753

[B38] Janetzki S Britten CM Kalos M Levitsky HI Maecker HT Melief CJ Old LJ Romero P Hoos A Davis MM "MIATA"-minimal information about T cell assays Immunity 2009 31 527 528 10.1016/j.immuni.2009.09.007 19833080 PMC3762500

